# Supplementary material for: Expression variation and covariation impair analog and enable binary signaling control
Source: Mol Syst Biol. 2018 May 14;14(5):e7997. doi: 10.15252/msb.20177997 (PMC5951153; doi:10.15252/msb.20177997)
Supplement: Supplementary file 1 — Expanded View Figures PDF [file MSB-14-e7997-s001.pdf]

# Expanded View Figures

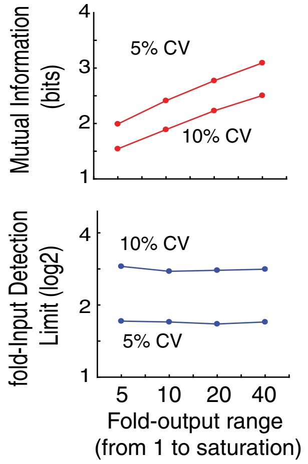


**Figure EV1. Comparison of fIDL and mutual information (MI) analysis.**

MI analysis requires a fold-output range which we added to the model in Fig 1A by using a saturation term for y4 (see Materials and Methods). As shown for CVs of 5 and 10%, in contrast to MI analysis (top), fIDL analysis (bottom) is largely independent of the fold-output range.

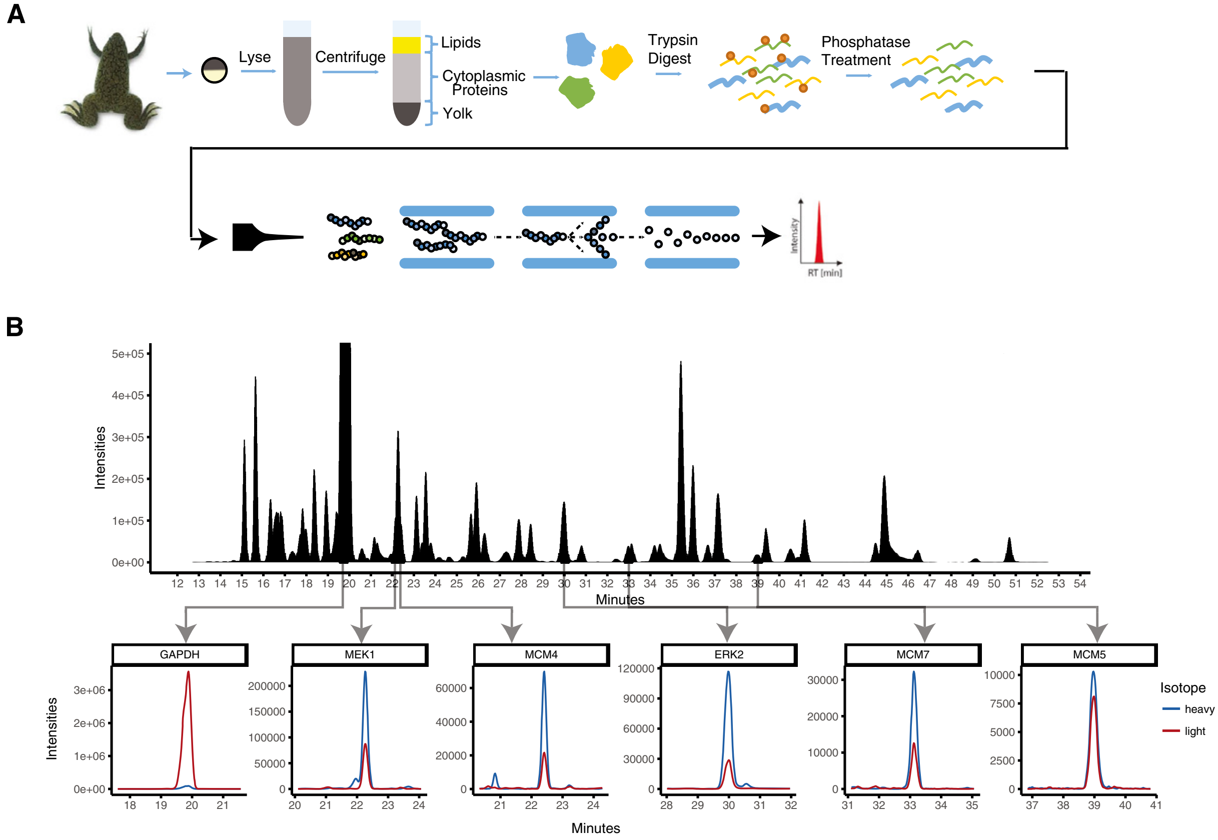


**Figure EV2. Selected reaction monitoring mass spectrometry approach to measure tens of proteins in parallel in single *Xenopus* eggs.**

A Schematic of protocol to quantitate the abundance of tens of endogeneous proteins in parallel in a single *Xenopus* egg.

B Typical selected reaction monitoring mass spectrometry (SRM-MS) chromatogram.

 © 2018 The Authors

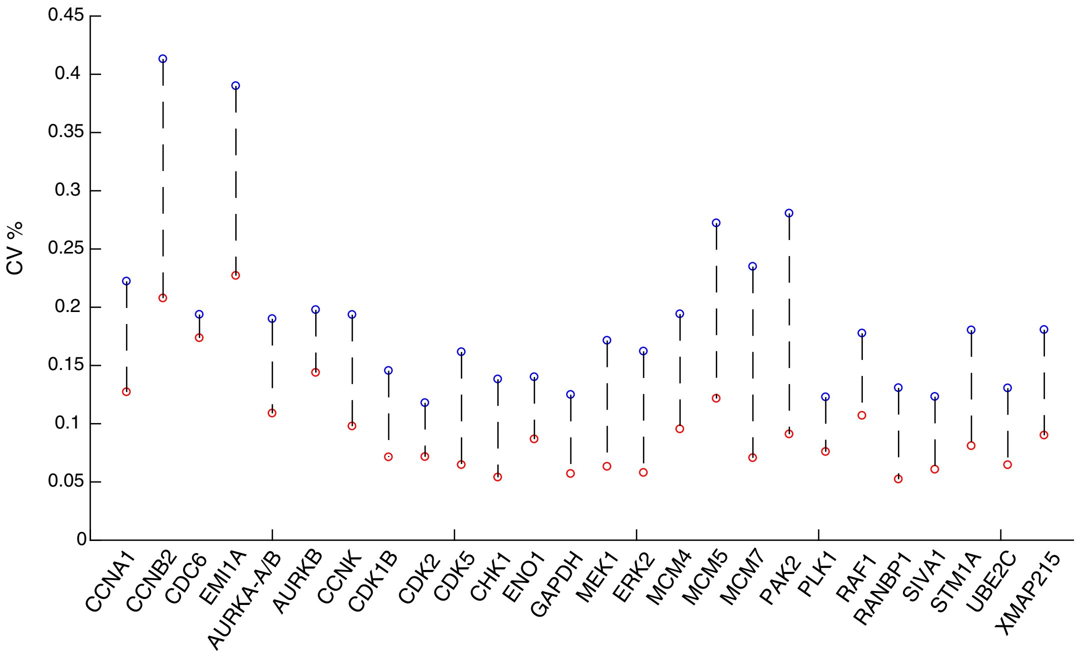


**Figure EV3. Bootstrap analysis of CVs of the relative abundance of 26 proteins using a 60-egg set collected at 60 min after egg activation.**

Bootstrapping of random samples was performed 2,000 times with replacement. We used the bootstrap analysis to determine CVs for the entire 60-egg dataset (blue circles) or on six batches of 10 eggs that were sequentially analyzed on the mass spectrometer (red circles). The lower CVs for batches of sequentially analyzed cells (median CV of 9% for the 26 proteins) argues that accurate concentration comparison using SRM analysis is optimally performed in batches of samples analyzed sequentially.

© 2018 The Authors

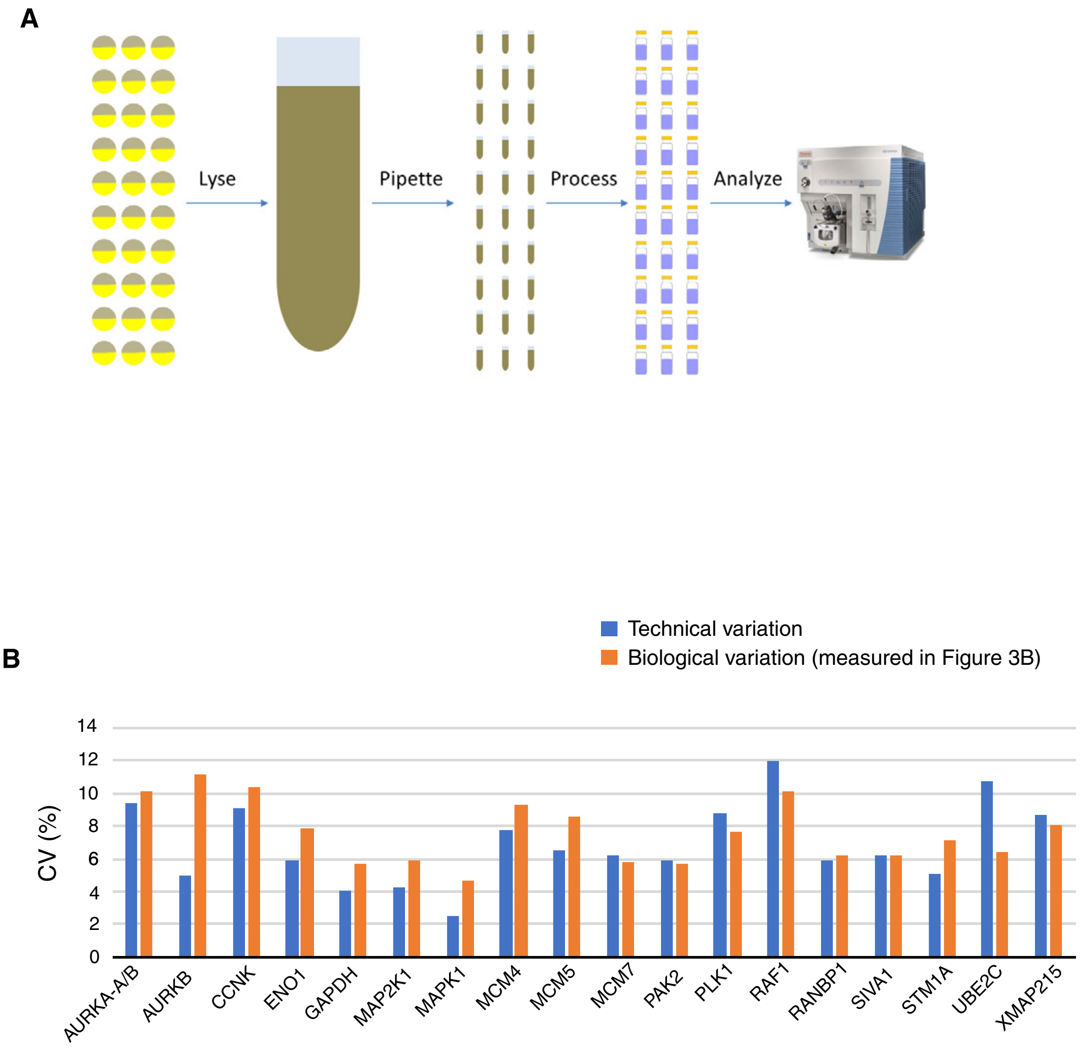


**Figure EV4. Comparison of technical and biological variation in the SRM mass spectrometry measurements.**

A Schematic of the experimental design. To measure technical variation, 30 individual eggs were lysed and mixed together to collapse any biological variability. The lysate mixture was then pipetted into 30 individual tubes and processed individually before carrying out SRM analysis to quantify sample handling variation.

B The CVs due to technical variation were compared to the CVs measured in Fig 3B.

 © 2018 The Authors

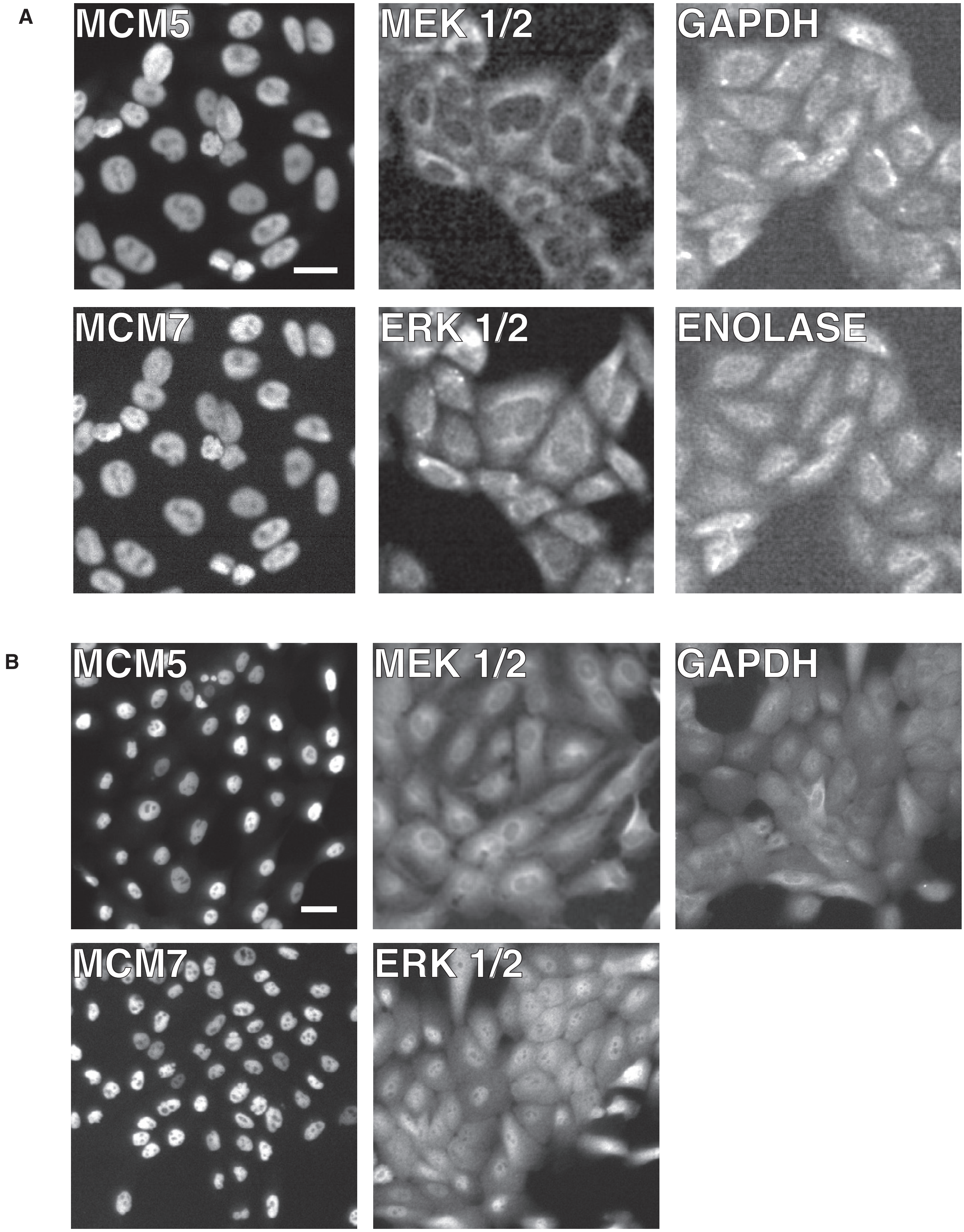


**Figure EV5. Representative images of immunohistochemistry staining of the proteins studied.**

A Images from HeLa cells. Scale bar is 20 μm.
B Images from MCF10A cells. Scale bar is 20 μm.

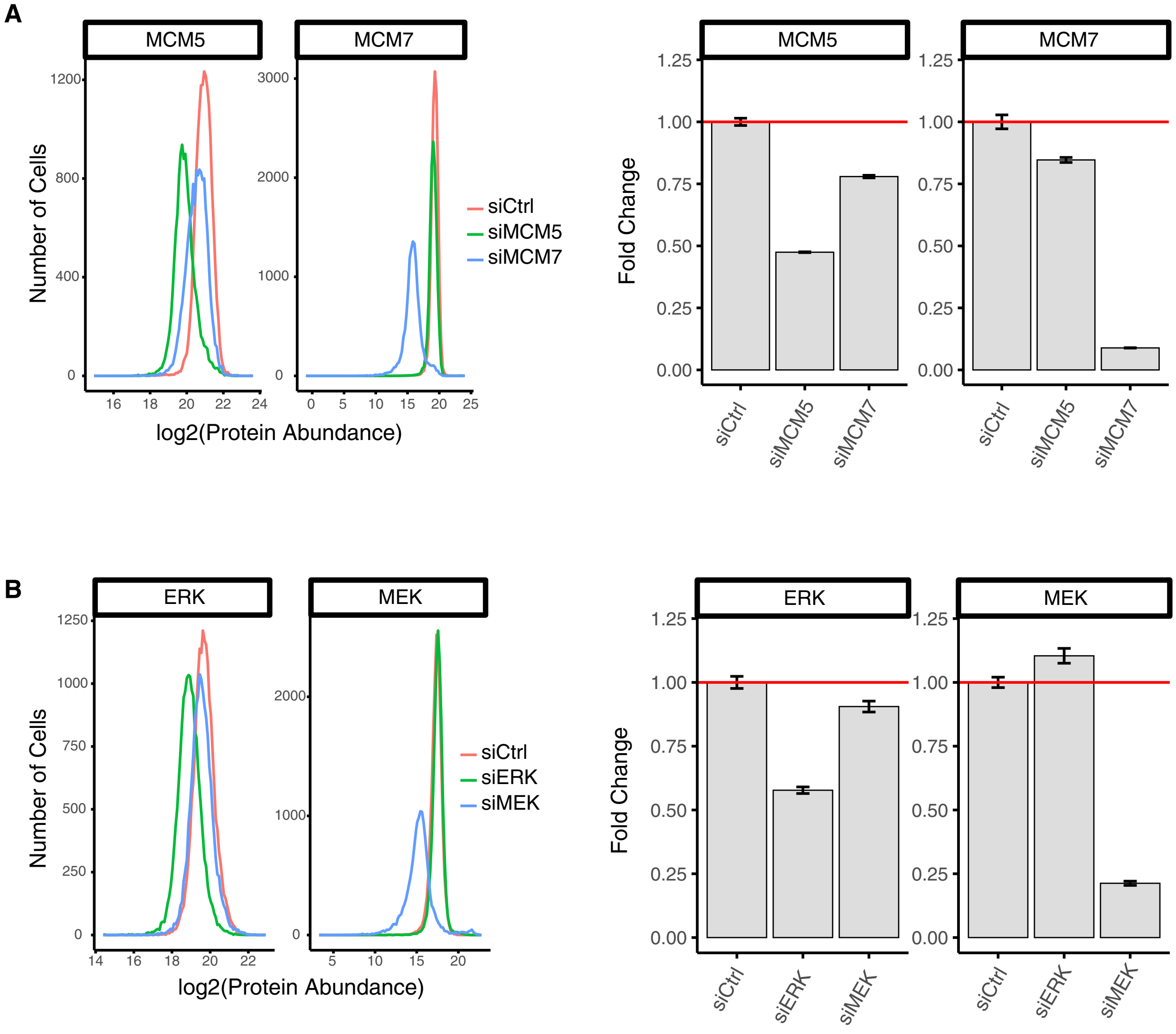


**Figure EV6. siRNA-mediated depletion experiments.**

A, B These experiments were carried out to validate the specificity of the respective antibodies and to test for co-regulation of protein expression of (A) MCM5/MC7M7 and (B) MEK/ERK. HeLa cells were transfected with the respective siRNA. Forty-eight hours later, the cells were fixed, stained, and imaged to quantify expression of the respective proteins. Approximately 1,000 cells were quantified in each histogram and barplot. Error bars show SEM. Results shown are representative of three independent experiments.


© 2018 The Authors
